# Tracking the evolutionary origins of dog-human cooperation: the “Canine Cooperation Hypothesis”

**DOI:** 10.3389/fpsyg.2014.01582

**Published:** 2015-01-15

**Authors:** Friederike Range, Zsófia Virányi

**Affiliations:** ^1^Comparative Cognition, Messerli Research Institute, University of Veterinary Medicine, Vienna, Medical University of Vienna, University of ViennaVienna, Austria; ^2^Wolf Science CentreErnstbrunn, Austria

**Keywords:** domestication, evolution, cooperation, attention, Canis lupus, Canis familiaris, tolerance

## Abstract

At present, beyond the fact that dogs can be easier socialized with humans than wolves, we know little about the motivational and cognitive effects of domestication. Despite this, it has been suggested that during domestication dogs have become socially more tolerant and attentive than wolves. These two characteristics are crucial for cooperation, and it has been argued that these changes allowed dogs to successfully live and work with humans. However, these domestication hypotheses have been put forward mainly based on dog-wolf differences reported in regard to their interactions with humans. Thus, it is possible that these differences reflect only an improved capability of dogs to accept humans as social partners instead of an increase of their general tolerance, attentiveness and cooperativeness. At the Wolf Science Center, in order to detangle these two explanations, we raise and keep dogs and wolves similarly socializing them with conspecifics and humans and then test them in interactions not just with humans but also conspecifics. When investigating attentiveness toward human and conspecific partners using different paradigms, we found that the wolves were at least as attentive as the dogs to their social partners and their actions. Based on these findings and the social ecology of wolves, we propose the *Canine Cooperation Hypothesis* suggesting that wolves are characterized with high social attentiveness and tolerance and are highly cooperative. This is in contrast with the implications of most domestication hypotheses about wolves. We argue, however, that these characteristics of wolves likely provided a good basis for the evolution of dog-human cooperation.

## Introduction

Cooperation is a fundamental aspect of human societies and has triggered much research in various fields like economics, psychology and biology. Although it is clear that human collaborative skills are exceptional, studying the cognitive and emotional processes of animal species that may underlie their cooperative interactions may reveal the evolutionary origins and the functional relevance of cooperation.

For a long time, the most common approach to investigate the evolutionary origin of human skills was to study non-human primates. More recently, building on the hypothesis that dogs and humans went through convergent evolution, researchers have suggested that dogs might be an additional, and in some respect, more informative model when investigating the evolution of human social behavior and cognition (Miklósi et al., 2004; Hare and Tomasello, 2005; Fitch et al., 2010). This idea is built on the assumption that dogs have been selected to cooperate and communicate with humans during domestication and, thus, evolved some genetic predispositions allowing them to develop skills shared with humans (Hare et al., 2002; Topál et al., 2009; Miklósi and Topál, 2013). Accordingly, it has been suggested that in a unique way, domestication has equipped dogs with two abilities necessary for cooperative problem solving—namely social tolerance and Social attentiveness, that enable them to adjust their behavior to that of their social partners (Ostojić and Clayton, 2014).

**Social tolerance**, i.e., allowing a potential partner to come close even around food, has been shown to be a prerequisite of cooperation in several animal species. For instance, it has been argued that bonobos (*Pan paniscus*) outperform chimpanzees (*Pan troglodytes*) in cooperative interactions (Hare et al., 2007) because they are less aggressive and more tolerant in a food sharing context than their closest relatives, chimpanzees (Hare et al., 2007; Hare and Kwetuenda, 2010; see also Petit et al., 1992 on similar results in Tonkean *(Macaca tonkeana)* and Rhesus macaques *(Macaca mulatta)*). Moreover, also at an individual level, tolerant individuals usually outperform less tolerant ones in cooperative tasks (marmosets (*Callithrix jacchus*): Werdenich and Huber, 2002; chimpanzees: Chalmeau and Gallo, 1993; Melis et al., 2006b; rooks (*Corvus frugilegus*): Seed et al., 2008; Scheid and Noë, 2010). Along the lines of this argument, Hare and Tomasello (2005) proposed that selection for a tamer temperament and for reduced fear and aggression explains the higher success of dogs in cooperative and communicative interactions with humans in comparison to wolves, the closest wild-living relative of dogs (emotional reactivity hypothesis). Recently, this hypothesis has been extended to suggest that during domestication dogs became less aggressive and more tolerant than wolves not just toward humans but also toward conspecifics (Hare et al., 2012, for an extensive discussion of the two versions of the emotional reactivity hypothesis see Virányi and Range (2014)).

KEY CONCEPT 1. Social tolerance Social tolerance refers to the close proximity of individuals (usually measured in the context of feeding), which is not accompanied with aggression or, if aggression occurs, it is bidirectional and ritualized. Across-species differences in social tolerance may rely on species-level differences in the underlying social emotions, but may also reflect more or less successful communication.

A second prerequisite of successful cooperation is **social attentiveness**, that is, paying sufficient attention to one's partners in order to adjust to their behavior and thus to cooperate (see for example studies using the loose string paradigm (Melis et al., 2006a,b; Seed et al., 2008; Péron et al., 2011; Plotnik et al., 2011)). Attentiveness toward potential partners, however, varies between species (Range et al., 2009a) and contexts (Range et al., 2009a) as well as according to the relationship the subject has with its partner (Range and Huber, 2007; Scheid et al., 2007; Horn et al., 2013). The link between attention and action coordination has so far mainly been studied in regard to social learning where several studies found that the amount of attention paid to potential models seems to be more or less directly linked to the success in acquiring specific behaviors (Lonsdorf, 2005; Ottoni et al., 2005; Renevey et al., 2013). Dogs have proven successful in several tasks that are thought to require high attention toward conspecifics and humans, such as experiments on social learning (Kubinyi et al., 2003; Topál et al., 2006; Range et al., 2007, 2011; Huber et al., 2009, 2014; Miller et al., 2009; Mersmann et al., 2011), social referencing (Merola et al., 2012a,b), communication (Virányi et al., 2004, 2006; Schwab and Huber, 2006; Udell and Wynne, 2008; Dorey et al., 2009; Kaminski et al., 2012), responding to unequal rewards (Range et al., 2009b, 2012) and cooperation (Naderi et al., 2001; Bräuer et al., 2013; Ostojić and Clayton, 2014). Furthermore, young dogs follow human pointing better and look at humans more readily than human-raised wolves (Miklósi et al., 2003; Gácsi et al., 2009). Consequently, it has been proposed that by means of positive (both evolutionary and ontogenetic) feedback processes, dogs have developed increased social attentiveness compared to wolves and thus, can achieve more complex forms of dog-human communication and cooperation than wolves (Miklósi et al., 2003; Virányi et al., 2008).

KEY CONCEPT 2. Social attentiveness Social attentiveness describes to what extent an individual pays attention to its companions and monitors their behavior and interactions. Obviously, it can greatly facilitate one's success in cooperating or competing with others or in gaining additional (and mostly highly relevant) information from their observation.

To sum up, these non-exclusive **domestication hypotheses** imply that wolves are less tolerant and less attentive than dogs. Importantly, however, all of the domestication hypotheses addressing the social skills of dogs and wolves rely on findings of experimental studies that compared the animals' interactions only with humans (Hare et al., 2002; Miklósi et al., 2003; Topál et al., 2005; Udell and Wynne, 2008; Virányi et al., 2008; Gácsi et al., 2009; Udell et al., 2011; Gácsi et la., 2013). Therefore, it is unclear if the few differences described so far reflect merely differences in the readiness of dogs and wolves to interact with humans or more fundamental differences regarding their social tolerance and social attentiveness; two prerequisites of cooperation. In the former case, we would expect that dogs and wolves show different behaviors only when interacting with humans but not conspecifics, simply because wolves are not quite as comfortable around humans as dogs and thus unable to fully concentrate on the human actions. In the latter case, however, we would expect dog-wolf differences also in other social contexts that do not involve humans. That is, in this case we would also expect differential social tolerance and/or attentiveness in interactions with conspecifics, as has been shown in other closely related species such as chimpanzees and bonobos or different macaque species as mentioned above.

KEY CONCEPT 3. Domestication hypotheses Domestication hypotheses propose evolutionary scenarios to explain the behavioral differences of dogs and wolves. Importantly, the adaptational demands that have presumably shaped the social cognition of the domestic dog are thought to have played an important role also during human evolution. Many of these hypotheses imply that wolves are socially less tolerant and less attentive than dogs.

## Our contributions

In order to better understand the effects of domestication on the social behavior of dogs, we think it is necessary to take a different approach, namely, rather than focusing purely on human-animal interactions, we should investigate to what extent and in what form the evolutionary precursors of dog social behavior can be found in wolves. To achieve this aim it is crucial to compare the interactions of dogs and wolves not only with humans, but also with conspecifics.

Obviously, living with or close to humans has certainly imposed important adaptational demands on the evolution of dog behavior (Miklósi and Topál, 2005; Topál et al., 2009); however, living in conspecific groups and interacting with other dogs has also always been part of the life of domestic dogs. Pet dogs represent a small part of the entire dog population with current estimates suggesting that free-ranging dogs represent about 76–83% of the global dog population (Hughes and Macdonald, 2013; Lord et al., 2013). These millions of dogs live more or less independently from humans, in conspecific groups in which their survival is greatly determined by successful communication and social maneuvering in intraspecific contexts (Bonanni and Cafazzo, 2014). Given this complex social ecology of the domestic dog, tracking the evolution of dog behavior and gaining a complete picture of the social competence of dogs and wolves require the study of various populations of both dogs and wolves as well as other canids that grew up and live in different social conditions (Udell et al., 2012; Miklósi and Topál, 2013).

**Figure 1:**
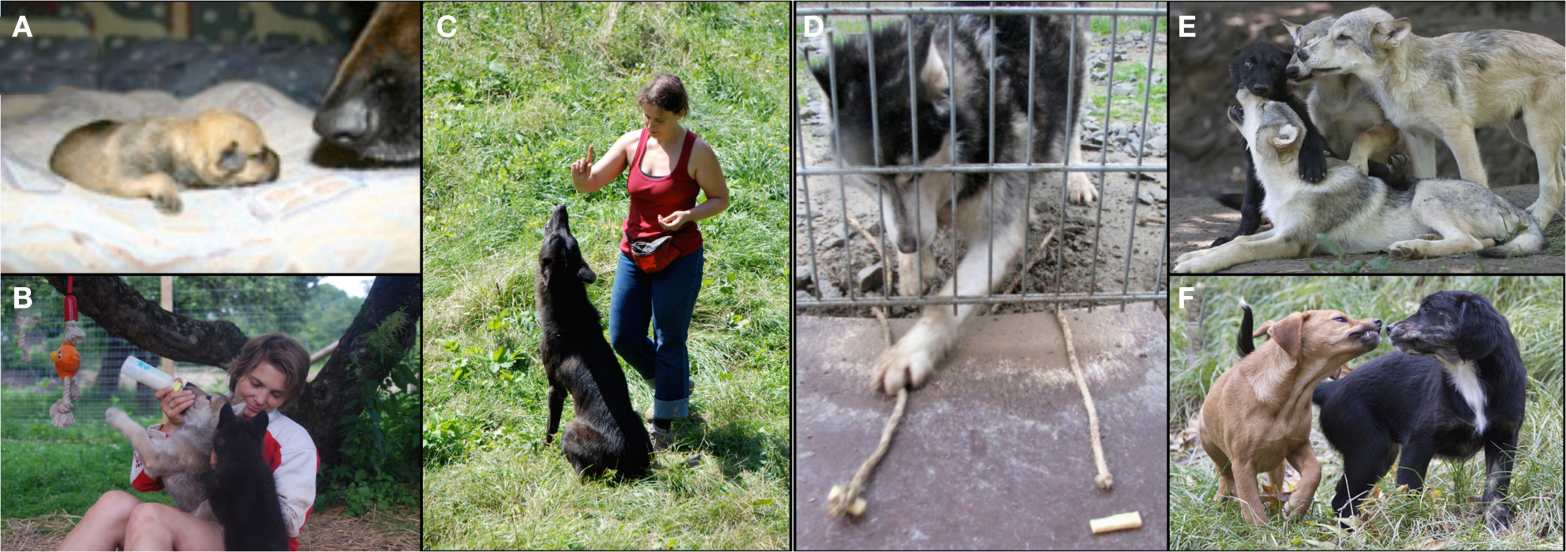
**Life at the Wolf Science Center**. Pictures show the socialization of the study animals with pet dogs **(A)**, hand raising of the animals **(B)**, interactions with humans **(C)**, participation in experiments **(D)** and pack life of wolves **(E)** and dogs **(F)** (Photographers: F. Range, F. Schwärzler, H. Möslinger, W. Vorbeck, P. Kaut).

Our contribution to this endeavor is to compare dogs and wolves that live in packs in large enclosures and are used to interacting with humans on a cooperative basis. For this aim, together with Kurt Kotrschal, we set up the Wolf Science Center that allows for testing adult wolves and dogs raised and kept in an identical way and socialized with humans and with conspecifics to a similar extent (Figure 1). Ensuring that wolves and dogs have the same experiences is important if we want to attribute observed dog-wolf differences to evolutionary changes rather than individual experiences. Pet dogs usually grow up in the human environment and thus have ample opportunities to learn how to interact and communicate with humans. On the one hand, these experiences can enhance their cognitive skills due to living in a much more complex social environment—an ontogenetic process that has been named “enculturation” (Call and Tomasello, 1996). On the other hand, having to interact and communicate with another species might greatly influence how attentive they are toward humans, which in turn affects their performance in social tasks involving humans (see also Miklósi et al., 2004; Topál et al., 2009). Thus, unless the animals grow up under identical conditions, not all dog-wolf dissimilarities should automatically be attributed to domestication, that is, assumed to rely on genetic changes that occurred since the dog separated from its closest wild-living relative, the wolf (Pang et al., 2009).

Our set-up is unique in comparison to earlier projects attempting to track evolutionary changes in canines (for an extensive review of these projects see (Virányi and Range, 2014)) in that (1) we can investigate not just the cognitive abilities of very young animals, but also of adult animals that have had similar experiences throughout their lives, (2) our set-up of keeping the animals in packs in large enclosures allows us to study their behavior with conspecifics under conditions that allow comparisons also with wild-living wolf and free-ranging dog packs, (3) their socialization enables us to explore the animals interactions with conspecifics as well as humans partly using the same experimental paradigms, and (4) we have an adequate sample size. At the Wolf Science Center we can study and compare human-animal as well as animal-animal interactions and can observe the animals' social behavior during spontaneous interactions within their packs.

So far, we have shown in several experiments that wolves pay as much attention to human partners as dogs do and that wolves can even outperform dogs in learning from observation of a conspecific, indicating the high social attentiveness of the species. We have also run experiments comparing their social tolerance toward conspecifics and humans, but this line of research is in progress and thus will not be discussed here in great detail.

**Figure 2:**
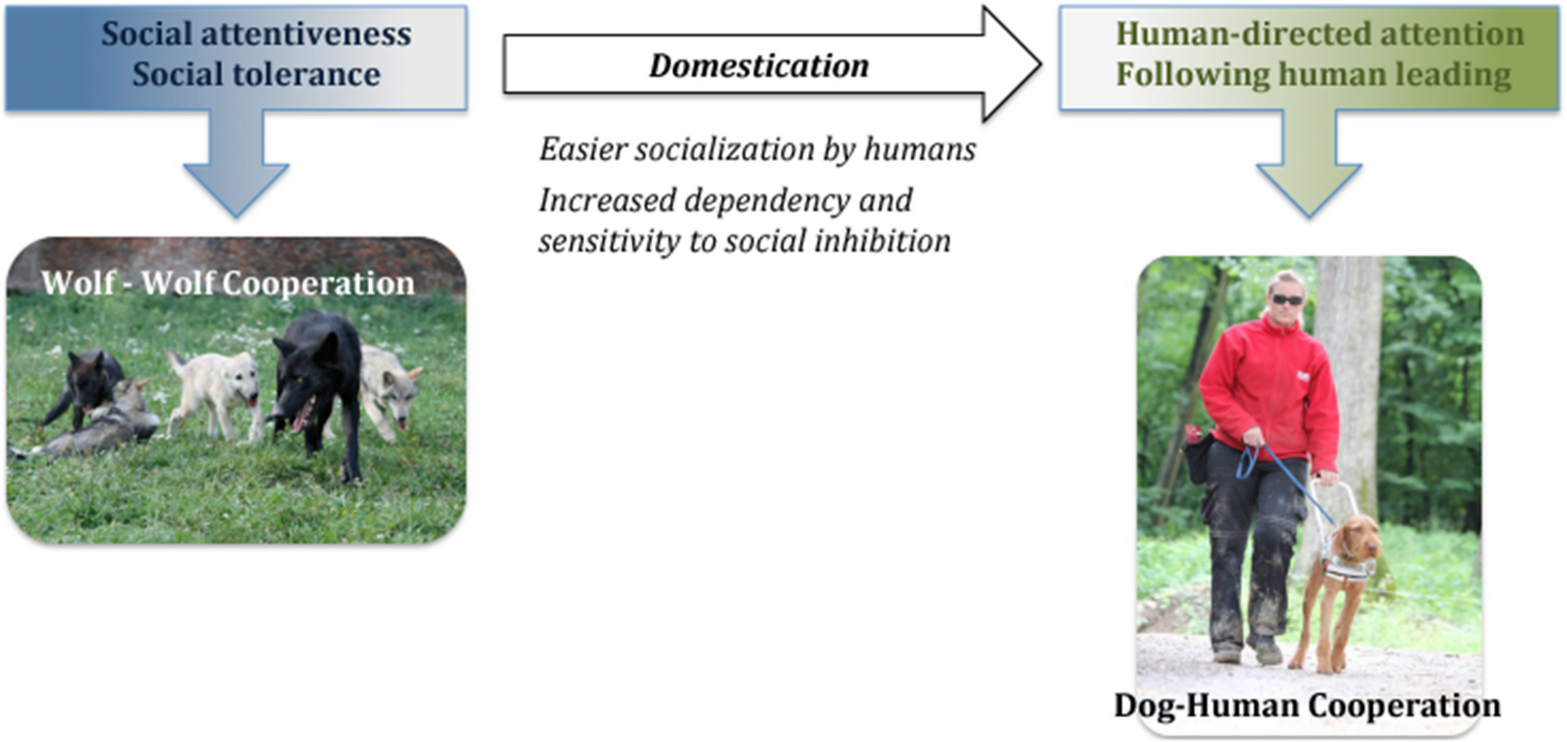
**Diagram of the “Canine Cooperation Hypothesis”**.

Importantly, the results of our work so far have shown that the implications of the current domestication hypotheses about the low social attentiveness and tolerance of wolves are incorrect. Therefore, we have proposed the ***Canine Cooperation Hypothesis*** (Range and Virányi, 2013, 2014; Virányi and Range, 2014) that postulates that wolf-wolf cooperation constitutes the basis also for dog-human cooperation and that no additional selection for social attentiveness and tolerance was necessary to allow for dog-human cooperation to evolve (Figure 2). Rather, the latter has probably been facilitated by dogs becoming able to more easily lose their fear of humans and be comfortable around them, which is obvious in the less intensive socialization needed by dogs to avoid fear of humans in contrast to wolves (Scott and Fuller, 1965; Klinghammer and Goodmann, 1987). In this review, we will outline our results on the social attentiveness of dogs and wolves that support the Canine Cooperation Hypothesis as well as some previous data collected on social tolerance.

KEY CONCEPT 4. Canine Cooperation Hypothesis Based on findings that in intraspecific contexts wolves are at least as socially attentive and tolerant as dogs, the Canine Cooperation Hypothesis postulates that dog-human cooperation evolved on the basis of wolf-wolf cooperation. In contrast to many domestication hypotheses, it suggests that dogs did not need to be selected for a general increase in their social attentiveness and tolerance.

### Monitoring human behavior

To test whether wolves and dogs that have been socialized to humans to an equal amount, pay attention to human actions, we tested the WSC dogs and wolves in a local enhancement task, where a demonstrator indicated the location of a food reward. Local enhancement is thought to offer a plausible explanation as to how animals might learn where to find food by paying attention to a place or location where a conspecific is showing a species-specific behavior and subsequently visiting that place (Galef and Giraldeau, 2001; Renevey et al., 2013).

**Figure 3:**
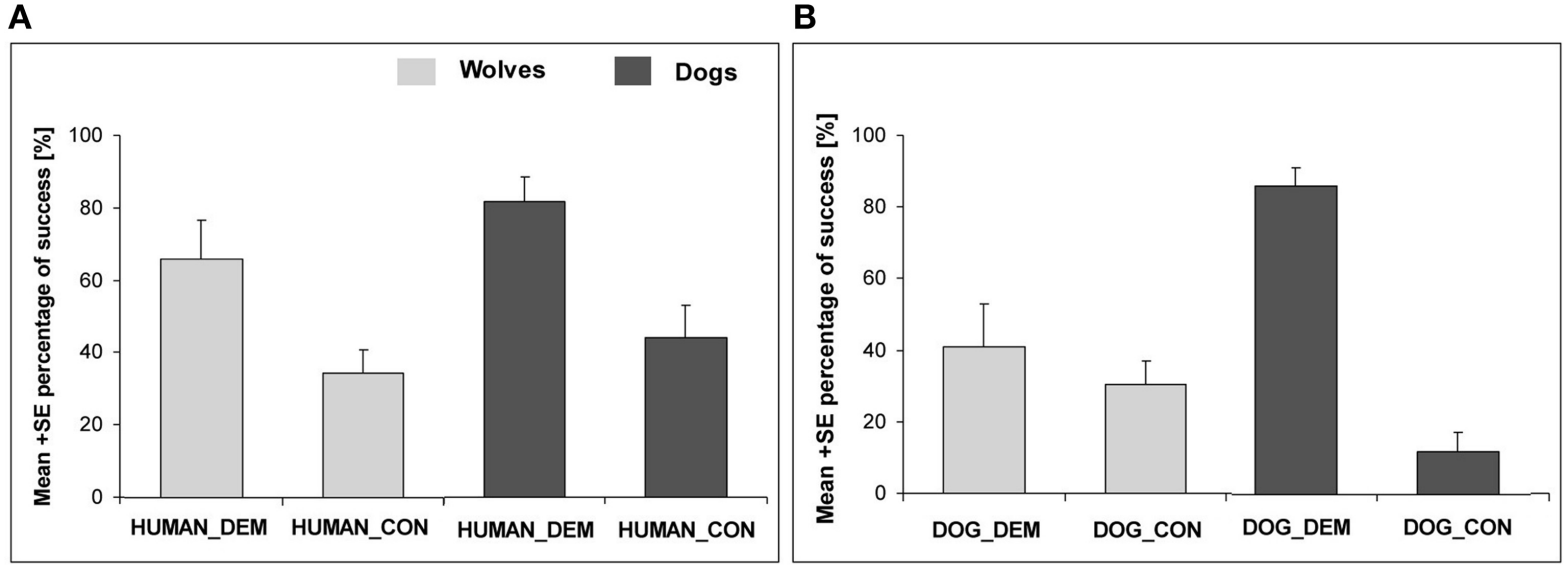
**Social learning from a human and conspecific demonstrator**. **(A)** depicts the success of wolves and dogs in the human demonstration (HUMAN_DEM) and respective control condition (HUMAN_CON). **(B)** shows the success of wolves and dogs in the conspecific demonstration (DOG_DEM) and control condition (DOG_CON). Adapted from Range and Virányi (2013).

In our study, the subjects observed a familiar human (hand-raiser) (1) hide a food reward (1-day-old dead chick) in one of three possible locations on a meadow or (2) pretend to hide a food reward (Range and Virányi, 2013). The second condition was carried out to investigate how much attention the subjects paid to the details of the demonstration, i.e., if they recognized that food was actually hidden or not. Finally, in a third, control (no demonstration), condition the food reward had been hidden before the subject was led onto the meadow to test if the animals could find the food reward without any demonstration, only relying on olfaction. In this no demonstration control both wolves and dogs were less successful in finding the chick than in the first condition, where a human demonstrator hid the chick. Interestingly, the dogs outperformed the wolves in both test and control conditions, suggesting that they relied more on their nose to find the hidden food than the wolves. Furthermore, when comparing the “with” or “without” chick demonstration trials, we found no difference between wolves and dogs when a human was the demonstrator: both groups clearly differentiated whether or not the human demonstrator actually hid a chick or only pretended to do so (Figure 3A). These results show that wolves can use the information provided by a familiar human and pay sufficient attention to their actions to solve such a local enhancement task.

Further support for this conclusion comes from another study where we investigated gaze following into distant space and around barriers in wolves (Range and Virányi, 2011). This ability to coordinate with others' head orientation to look in the same direction is considered a key step toward an understanding of others mental states like attention and intention (Baron-Cohen, 1995; Tomasello et al., 2005) and thus, is potentially also very important for being able to successfully cooperate. However, while gaze following into distant space could be simply a socially facilitated orientation response (i.e., a predisposition to look where others are looking) (Povinelli and Eddy, 1996), gaze following around barriers, where individuals need to reposition themselves to look behind the obstacle and assess the visual persepctive of the cue-giver different from their own, has been suggested to require a mental representation of the looker's visual perspective (Povinelli and Eddy, 1996) or learning how visual barriers impair perceptions (Tomasello et al., 1999). Accordingly, this latter ability to track another's gaze around obstacles seems to be cognitively more advanced, and has been suggested to occur especially in species with high levels of cooperative and competitive interactions (Schlögl et al., 2007). Our results showed that wolves followed human gaze as readily as conspecific gaze implying their high social attention and their readiness to accept humans as social partners who might provide important information.

However, the fact that wolves, when given intensive human socialization, accept humans as social partners and can use human-given information as well as dogs do, does not necessarily mean that domestication had no effect on dog-human cooperation. Instead, it is possible that domestication in dogs did alter these skills but in wolves intense socialization with humans provides an alternative route of acquiring them; for instance taking their natural fear of humans away may enable wolves to use their species-specific cognitive abilities also when interacting with humans (see Introduction, Udell et al., 2008).

### The social ecology of wolves

Both social learning and gaze following are likely to be adaptive in wild-living wolves in several contexts (see for example, Thornton and Clutton-Brock, 2011). Wolves are cooperative breeders that rely on supporting each other not just when raising pups, but also in territorial defense and when hunting large game or defending their kills (Mech, 1970; Mech and Boitani, 2003; Kaczensky et al., 2005). This overall high dependency on cooperative interactions with conspecifics requires wolves to pay close attention to others in order to coordinate their actions with each other and probably some social learning to be able to learn the necessary skills for successful cooperation. For example, it has been proposed that wolf pups learn where to find prey, how to kill it and how to avoid injuries by accompanying their parents during hunting excursions and socially learning from them (Packard, 2003). Consequently, we propose that due to their social organization and their high dependency on cooperation, several emotional and cognitive characteristics that have been proposed to characterize dogs may already be present in wolves (Derr, 2011; Kotrschal, 2012; Range and Virányi, 2014). In other words, it is important to examine to what extent and in what format we can find the evolutionary precursors of dogs' social behavior in wolves. Since we have the best chance to detect such social capabilities of wolves in intraspecific contexts, we need to investigate the behavior of our wolves and dogs also in relation to conspecifics.

### Monitoring the behavior of conspecifics

In the local enhancement study described in the previous section, we did not just test our animals with a human demonstration, but also with a dog demonstration. The subjects, both wolves and dogs, had established close relationships with the demonstrator dogs during the hand-raising period. They readily greeted them, played with them, and established dominance relationships with them, and all wolves and dogs readily submitted to the demonstrator dogs suggesting that they were perceived as conspecifics. In the test conditions, the subjects observed (1) the conspecific carrying and dropping a food reward (1-day-old dead chick) on one of the three paths, or (2) the conspecific walking out on a path without a food reward. These two conditions were interspersed with the three conditions mentioned above (human demonstrator hiding the food, human demonstrator pretending to hide food, control without demonstration) in an order counterbalanced across and within subjects. Interestingly, comparing the control condition with the food hiding demonstrations (human and conspecific), we found that both wolves and dogs benefitted from a demonstration independent of the demonstrator species. However, when comparing the “with” and “without” chick demonstration trials, we found a difference in contrast to the results with the human demonstrator: while the dogs showed the same behavior after a conspecific demonstration as after human demonstration, in general the wolves paid less attention to the dog in the “with” and “without” chick demonstrations than the dogs did, and wolves did not differentiate between these two trials (Figure 3).

These last results are intriguing, since the question arises why the wolves showed special attention to the human demonstrations and clearly differentiated between “with” and “without” chick demonstrations, but paid less attention to the conspecific demonstration independently of the presence of reward. There are two explanations that we think likely, although they need further testing: (1) Since our wolves have a very cooperative relationship with the hand-raisers, usually being rewarded for attention during the daily training sessions, they also expected to get food in these test trials. In contrast, the wolves did not expect the conspecific to share food with them and thus it was less interesting to pay close attention to them, or (2) although the demonstrator dogs were trained to execute the demonstration, they disliked the chick used as food reward. It is possible that the wolves recognized this dislike due to increased attention to the behavioral details of the conspecific models in comparison to the dogs. Assuming that wolves, similarly to tamarins *(Saguinus oedipus)* (Snowdon and Boe, 2003), are sensitive to a display of disgust and can adjust their behavior accordingly, the behavior of the demonstrators might have decreased the interest of the wolves in finding the food reward.

**Figure 4:**
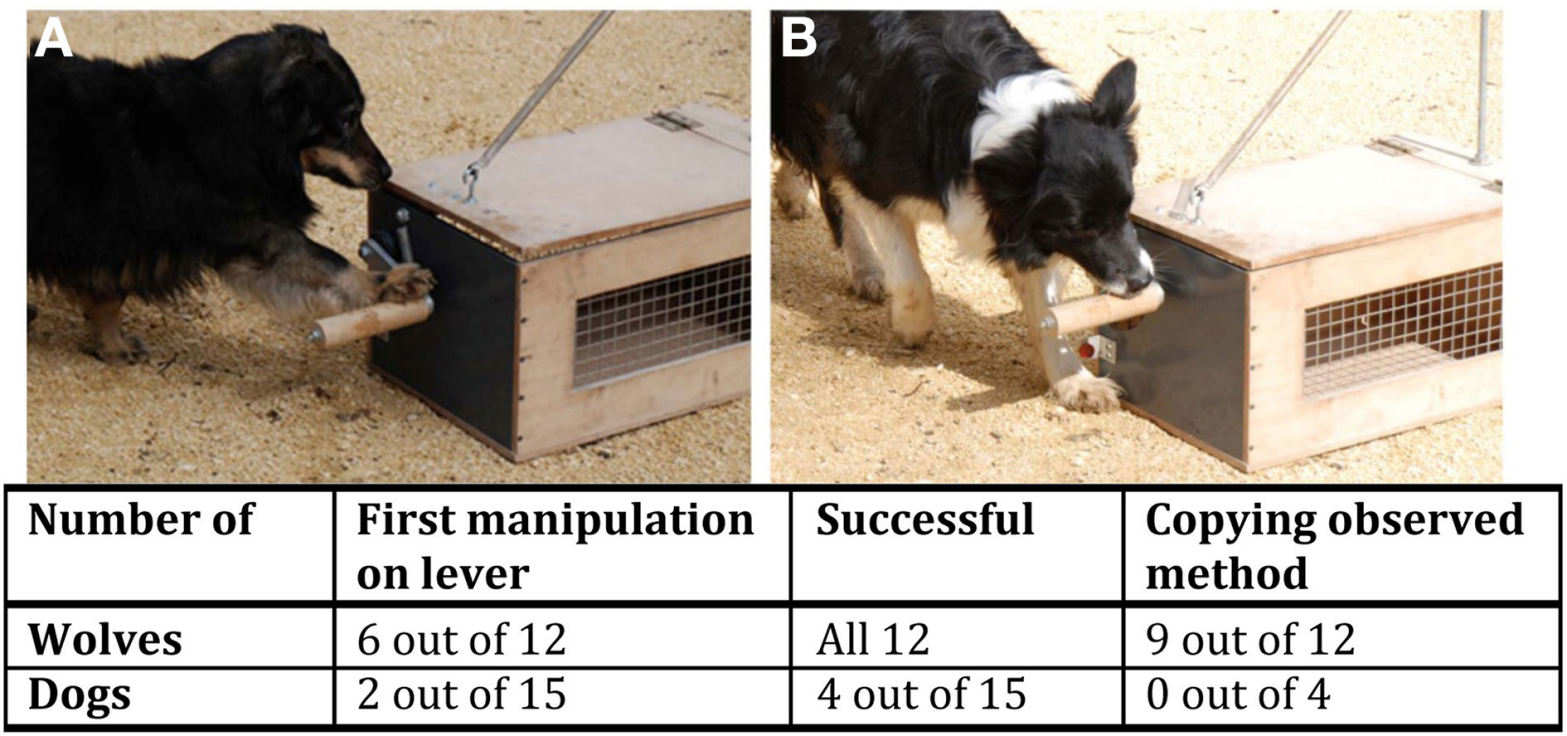
**Imitation of conspecifics**. The pictures depict the two different demonstrations (**A**: Paw demonstration; **B**: Mouth demonstration) of opening the baited box, wolves and dogs observed six times before being allowed to try to open the box by themselves. The table lists the number of animals that first manipulated the most relevant part of the box, the lever, that were successful at least once in opening the box and finally used the method that they had observed to open the box. In all three variables the wolves and dogs differed significantly from each other (for further details see Range and Virányi, 2014).

Both of these latter two speculations support the idea that wolves are very attentive toward behaviors of their social partners and maybe even more so than dogs. This idea is also supported by another social learning experiment, where we tested 6-months-old wolves and same aged dogs in a two-action imitation task following a conspecific demonstration (Range and Virányi, 2014). The subjects observed one of two familiar pet dogs opening a novel box using either its paw (Figure 4A) or its mouth (Figure 4B) to reach a food reward hidden inside. After each of six demonstrations, the observers were allowed to retrieve a piece of food from the box opened by the demonstrator. Afterwards, the subjects were released to manipulate the baited apparatus to see if and how they solved the task and whether they matched their behavior to the demonstrated action. We found that wolves clearly outperformed the dogs with all wolves opening the box at least once by actively manipulating the lever, while only 4 dogs out of the 15 were successful (Figure 4). Regarding imitation, we found that wolves significantly matched the demonstrated action when manipulating the box for the first time, whereas dogs used the two methods randomly. Moreover, wolves and dogs differed significantly from each other in regard to whether or not they matched the demonstrated action during their successful manipulation of the box with 9 of the 12 wolves matching the observed method, while none of the dogs did so.

It is possible that instead of trying to solve the problem by themselves, the dogs might have relied on the human experimenter to solve the task as has been shown in other experiments (Topál et al., 1997; Passalacqua et al., 2011; Horn et al., 2012) which might have masked their imitative abilities. However, our dogs first tried to open the box by themselves (we found no difference in the latency of wolves and dogs to manipulate the apparatus) and they approached a human only later, at a time when the wolves had already solved the problem, which suggests that this explanation is unlikely. Moreover, conducting control experiments, we could rule out that the difference between wolves and dogs arose due to differential developmental pathways of the two species or better physical insight of wolves. Rather it seems that the wolves were more sensitive toward the details of the action of a conspecific partner compared to the dogs, enabling them to solve this task.

To sum up, so far we have shown that wolves pay close attention to details of their pack-members' behavior when following their gaze or when imitating their action in a manipulative, two-action task. Moreover, since these animals are well-socialized with humans, they also follow human gaze into distant space, and profit from a human demonstration in a local enhancement task. Accordingly, wolves seem to possess at least one skill that has been suggested to be a precondition of successful cooperation, namely high social attentiveness toward social partners.

### Social tolerance

But how about the second skill proposed to be important for successful cooperation, social tolerance toward group members? Earlier observations of wolves and dogs raised and kept comparably, suggest that malamute puppies show earlier and more intense aggression than wolves (Frank and Frank, 1982). These observations were also confirmed in older animals, where dogs were overly aggressive in agonistic interactions and more so than wolves (Feddersen-Petersen, 1991, see Virányi and Range, 2014 for a thorough review of these data). In a first study investigating intra-species aggression and tolerance in our animals, we tested each animal with every other pack member in pair-wise food competition tests. We found that in dogs the higher-ranking member of each dyad monopolized the food resource, feeding alone most of the time and showed more aggression than the lower-ranking partner, whereas in wolves we did not find such an effect of rank position (Range et al. in revision). Moreover, the wolves co-fed longer than the dogs. In conjunction with Feddersen-Petersen's and Franks's observations, our data suggested that, at least in captivity, dogs form a steeper dominance hierarchy than wolves, which probably dissuades lower-ranking animals from challenging higher-ranking partners.

Similar comparisons have been conducted also in other closely related species, such as in macaques, for instance. Based on their aggression, tolerance, conciliatory behavior, dominance gradient and kin-bias, Thierry (2000) arranged the macaque species according to a four-grade scale. The first grade is characterized by unidirectional aggression of dominant animals with high and severe biting rates and subordinates generally fleeing or submitting when attacked. The species belonging here are characterized as having a steep dominance gradient and a low tolerance level. On the other extreme of the scale, the intensity of aggression and the biting rate are low, and most aggressive interactions are bidirectional, meaning that the victim of aggression protests or counter-attacks. In these species, the dominance gradient is less steep and tolerance is high. Thus, while the asymmetry of contests and the dominance gradient decrease from the first to the forth grade, social tolerance increases. Importantly, however, the dominance gradient is a characteristic of linear hierarchies and thus a low gradient does not imply that there is no linear and stable hierarchy. Since, as described earlier, the intraspecific social life of dogs has always been and is still highly relevant for the evolution of dog behavior, we suggest that comparing intraspecies aggression and tolerance in dogs and wolves can and should be integrated into such broader comparative and ecological frameworks. If adopting Thierry's (2000) scale to the dogs and wolves, the dog would be characterized as a less tolerant species than the wolf.

### Intraspecific cooperation in dogs

If attentiveness and tolerance are indeed relevant prerequisites for cooperation, we would expect that differences in those fundamental abilities will correlate with the degree of cooperativeness in various social contexts. As discussed earlier, the social life of wolves is centered on cooperation with their kin. In contrast to wolves, feral dogs do not live in family units but rather as multi-male/multi-female groups of largely unrelated individuals. And although they display differentiated social relationships with each other (Bonanni et al., 2010; Cafazzo et al., 2010), they do not breed cooperatively (Lord et al., 2013). Instead, female feral dogs mainly raise their pups alone (Daniels and Bekoff, 1989; Boitani and Ciucci, 1995) with fathers defending the pups occasionally. Moreover, dogs usually feed on stable food resources provided by humans (e.g., scavenging at rubbish dumps, or food provisioned by humans; Schmidt and Mech, 1997; Butler et al., 2004) and reports of group hunting in free-ranging dogs are rare (Butler et al., 2004; but see also Manor and Saltz, 2004). Consequently, dogs do not seem to be as cooperative as wolves with conspecifics, which has also been observed in captive settings (e.g., Feddersen-Petersen, 2007; but see Ostojić and Clayton, 2014).

## Conclusions

In conclusion, our own and previous data support the Canine Cooperation Hypothesis (Range and Virányi, 2013, 2014; Virányi and Range, 2014) that argues that the high social attentiveness, tolerance and presumable cooperativeness in wolves provided a good basis for dog-human cooperation to evolve. Our results contradict most domestication hypotheses that argue that in comparison to wolves, dogs were selected for increased attentiveness and tolerance. The Canine Cooperation Hypothesis is, however, compatible with other evolutionary theories emphasizing the role that wolf-human similarities in sociability and cooperativeness played in the evolution of dogs (Schaller and Lowther, 1969; Clutton-Brock, 1984; Schleidt, 1998) and with the domestication hypotheses that specifically address the human-directed behavior of dogs. For instance, it has been suggested that dogs were selected for reduced fear of and easier socialization with humans (Scott and Fuller, 1965; Klinghammer and Goodmann, 1987); this certainly helped them to extend their relevant social skills to interactions with humans—skills inherited from wolves. Further research needs to clarify, however, whether indeed only this easier socialization with humans differentiates dogs and wolves, but otherwise dog-human and wolf-wolf cooperation rely on similar mechanisms or if domestication has resulted also in other changes in the dogs' behavior relevant to their interactions with humans.

### Conflict of interest statement

The authors declare that the research was conducted in the absence of any commercial or financial relationships that could be construed as a potential conflict of interest.
